# Regulation of the *Formyl Peptide Receptor 1 (FPR1)* Gene in Primary Human Macrophages

**DOI:** 10.1371/journal.pone.0050195

**Published:** 2012-11-21

**Authors:** Claudio Gemperle, Mattia Schmid, Magdalena Herova, Jacqueline Marti-Jaun, Sophia J. A. Wuest, Christa Loretz, Martin Hersberger

**Affiliations:** 1 Division of Clinical Chemistry and Biochemistry, University Children’s Hospital Zurich, Zurich, Switzerland; 2 Children’s Research Center, University Children’s Hospital Zurich, Zurich, Switzerland; 3 Center for Integrative Human Physiology, University of Zurich, Zurich, Switzerland; Duke University Medical Center, United States of America

## Abstract

The formyl peptide receptor 1 (FPR1) is mainly expressed by mammalian phagocytic leukocytes and plays a role in chemotaxis, killing of microorganisms through phagocytosis, and the generation of reactive oxygen species. A large number of ligands have been identified triggering FPR1 including formylated and non-formylated peptides of microbial and endogenous origin. While the expression of FPR1 in neutrophils has been investigated intensively, knowledge on the regulation of FPR1 expression in polarized macrophages is lacking. In this study we show that primary human neutrophils, monocytes and resting macrophages do express the receptor on their cell surface. Polarization of macrophages with IFNγ, LPS and with the TLR8 ligand 3M-002 further increases FPR1 mRNA levels but does not consistently increase protein expression or chemotaxis towards the FPR1 ligand fMLF. In contrast, polarization of primary human macrophages with IL-4 and IL-13 leading to the alternative activated macrophages, reduces FPR1 cell surface expression and abolishes chemotaxis towards fMLF. These results show that M2 macrophages will not react to triggering of FPR1, limiting the role for FPR1 to chemotaxis and superoxide production of resting and pro-inflammatory M1 macrophages.

## Introduction

The formyl peptide receptor 1 (FPR1) belongs to a family of G protein-coupled pattern recognition receptors, which are mainly expressed by mammalian phagocytic leukocytes and are key players in innate immunity and host defence [1], [2], [3]. In neutrophils, signalling through the FPR1 receptor plays a role in chemotaxis, killing of microorganisms through phagocytosis, and generation of reactive oxygen species [2]. In addition, FPR1 is thought to play a role in sensing of endogenous signals of dysfunctional cells, which should attract leukocytes to the site of inflammation and tissue damage [2].

The first ligands identified for FPR1 were the N-formylated peptides from bacteria, however, later it was found that such N-formylated peptides could also derive endogenously from mitochondria, released as a result of severe cell dysfunction or cell death [1]. However, it is currently not clear whether these mitochondria derived N-formylated peptides are produced *in vivo* [2]. In addition, a large number of microbial and endogenous peptides of various compositions have been identified as agonists for FPR1. These include formylated and non-formylated peptides of microbial and endogenous origin, like the GP-41 envelope protein of the human immuno-deficiency virus (HIV), annexin-1, and a list of peptides from peptide libraries [2], [4].

Several intracellular signalling pathways regulating chemotaxis and superoxide production are triggered by FPR1 in neutrophils [2]. Chemotaxis towards pathogens is mediated by activated Gα_i_ involving the PI3K family of kinases [5], [6], while several signalling pathways have been identified for superoxide production necessary for the oxidative burst. Superoxide production was shown to be transduced by PI3K mediated signalling pathways but also Rac [7] and PKC dependent pathways [8] are involved in superoxide production upon FPR1 triggering.

In addition, the receptor is regulated by desensitization upon activation with ligands [9]. After stimulation of the FPR1 receptor, the cellular responses rapidly decline in intensity and the cells become refractory to subsequent stimulations with the same agonist. This mechanism common to G protein-coupled receptors results at least partially from phosphorylation of the agonist-occupied receptor by G protein-coupled receptor kinases, leading to its internalization [10], [11].

FPR1 is highly expressed in myeloid cells like neutrophils, monocytes and macrophages [12] and the promoter region of the gene has been characterized identifying a myeloid specific transcription factor necessary for transcription of the gene [13]. While the expression of FPR1 in neutrophils has been investigated intensively, knowledge on regulation of FPR1 expression in polarized macrophages is lacking. In this study we investigate the regulation of FPR1 expression and function in primary human macrophages. We show that most polarized M1 macrophages do express FPR1 on their cell surface, while IL-4 and IL-13 polarized M2 macrophages do not have functional FPR1 cell surface expression.

**Figure 1:**
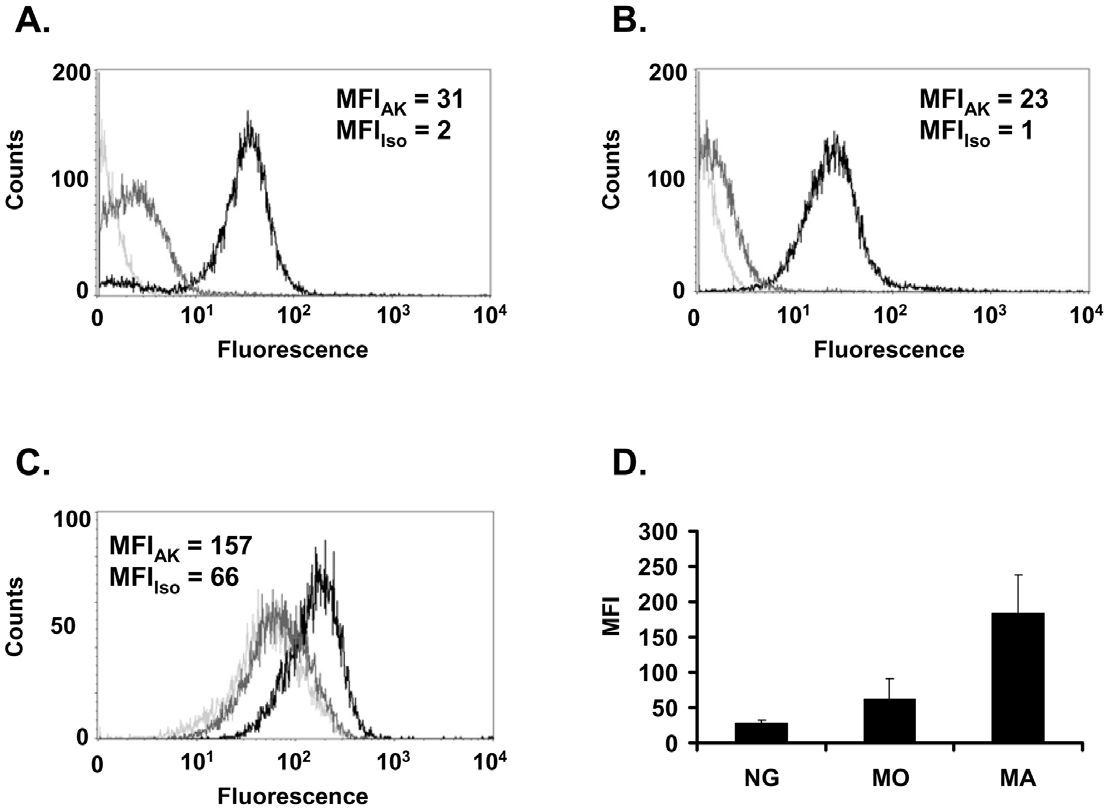
FPR1 cell surface expression on human neutrophils, monocytes, and macrophages. FACS analysis was performed to investigate cell surface expression of FPR1. A) Neutrophils. B) Monocytes. C) 9 day old macrophages. Autofluorescence of the cells is shown in light grey, the isotype control in grey and cells labeled with FPR1 Ab in black. D) Quantitative representation of the FPR1 median fluorescence intensity (antibody MFI minus isotype MFI) NG: neutrophils (n = 3). MO: monocytes (n = 5). MA: 9 day old macrophages (n = 9).

**Figure 2:**
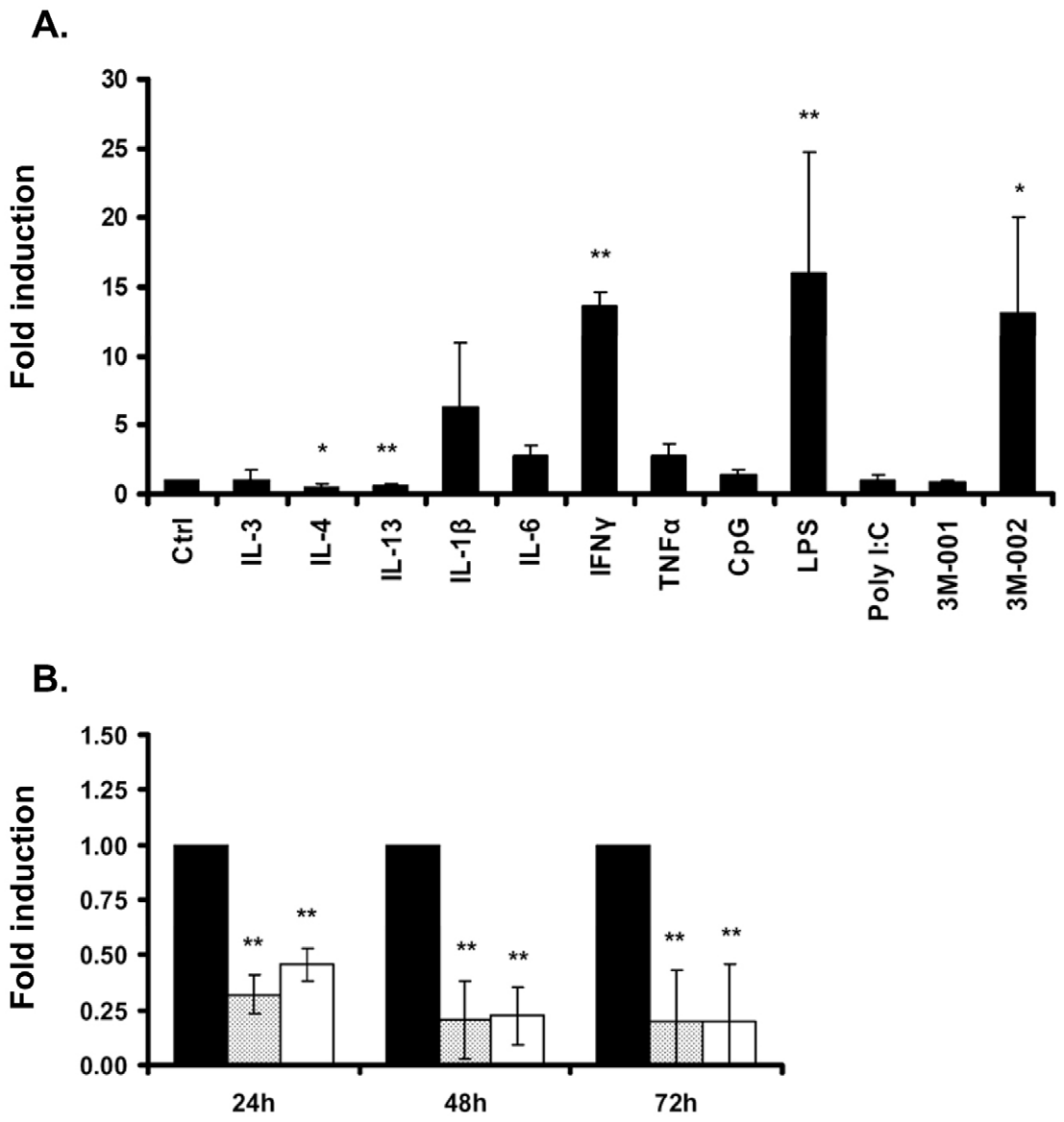
Regulation of FPR1 mRNA expression in human macrophages. A) Relative mRNA expression of FPR1 after stimulation of human macrophages with different stimuli for 24 hours. IL-3 (20 ng/ml), IL-4 (10 ng/ml), IL-13 (10 ng/ml), IL-1β (5 ng/ml), IL-6 (10 ng/ml), INFγ (50 ng/ml), TNFα (1 ng/ml), CpG (100 ng/ml), LPS (100 ng/ml), Poly I:C (1 ng/ml), 3M-001 (3 µM), 3M-002 (3 µM). B) Time-course experiment of FPR1 mRNA expression in macrophages stimulated with IL-4 (10 ng/ml) (dotted) and IL-13 (10 ng/ml) (white) for 24, 48 and 72 hours. The values are normalized for GAPDH mRNA expression and are presented relative to non-stimulated control macrophages (black). Bars display the mean and the standard deviation (±SD) of three independent experiments. *p<0.05, **p<0.01.

## Materials and Methods

### Material

The recombinant human cytokines IL-1β, IL-3, INFγ, and TNFα as well as lipopolysaccharide (LPS) and the Toll-Like Receptor (TLR) 3 agonist Poly I:C, were purchased from Sigma-Aldrich (Buchs, Switzerland). IL-4, IL-6, IL-13 were obtained from R&D Systems Europe Ltd. (Abingdon, United Kingdom). The TLR9 ligand, CpG-oligodeoxynucleotides (CpG), was synthesized by Microsynth (Balgach, Switzerland). The TLR7 and TLR8 ligands 3M-001 and 3M-002, respectively, were purchased from 3M Pharmaceuticals (St. Paul, MN, USA). FPR1 antibody (Formyl peptide receptor 1, anti-human, monoclonal antibody) was purchased from R&D Systems Europe Ltd.

### Preparation of Human Peripheral Monocytes and Cell Culture

White blood cells from healthy blood donors were isolated from commercially available and anonymized buffy coats (Blutspendezentrum Zurich, Schlieren, Switzerland) using Histopaque–1077 gradient (Sigma-Aldrich). Peripheral human monocytes were purified by capturing with anti-CD14 antibodies coupled to MACS microbeads (Miltenyi Biotech, Bergisch Gladbach, Germany). Monocytes were seeded with a density of 0.7*10^6^ cells/ml and were cultured for differentiation into macrophages for 7 days at 37°C and 5% CO_2_ in RPMI-1640 (Sigma-Aldrich) supplemented with 5% Fetal Calf Serum (Bioconcepts, Allschwil, Switzerland), 5% Human AB Serum (Sigma-Aldrich) and 1% Penicillin/Streptomycin (Invitrogen, Zug, Switzerland). For the stimulation experiments with the set of cytokines, the cells were washed once with PBS (Bioconcept) and incubated in RPMI-1640 (Sigma-Aldrich) supplemented with 5% Human AB Serum (Sigma-Aldrich), 1% Penicillin/Streptomycin (Invitrogen) and the indicated cytokines for different periods.

### Quantitative Real-Time PCR (qPCR)

Total amount of RNA was extracted using RNeasy Mini Kit (Qiagen AG, Hombrechtikon, Switzerland) and the reverse transcription reaction was performed with 0.5 µg RNA in a 20 µl reaction using random primers (Invitrogen) with the Superscript III Reverse Transcriptase (Invitrogen) according to the manufacturer’s instructions. The qPCR reaction was done on a LightCycler 480 system (Roche Diagnostics, Rotkreuz, Switzerland) utilizing the hot-start SYBR green method with the following parameters: preheating for 10 min at 95°C, followed by 45 cycles of denaturation for 5 sec at 95°C, annealing for 10 sec at 60°C and extension for 6 sec at 72°C. The quantitative PCR included 50 ng cDNA, 0.5 µM forward and 0.5 µM reverse primer and 5× SYBR green master mix (Roche Diagnostics). Primers were designed using the OLIGO 6.0 software (Molecular Biology Insights, Inc., Cascade, USA) (sequences are listed in Table S1).

### FACS Analysis

FACS analysis was performed using a monoclonal PE-labeled anti-human FPR1/FPR antibody (Ab) and an IgG2a isotype control (both R&D Systems, Minneapolis, MN, USA). As a positive control for the stimulations of the cells, CD206 and CD80 Ab were used (BD Biosciences, San Jose, CA, USA). Briefly, cells were resuspended in PBS containing 2.5% FCS and incubated in the dark for 30 min at 4°C before analysis on a FACS Calibur Analyzer (BD Biosciences, San Jose, CA, USA).

### Chemotaxis Assay

A total of 10^5^ macrophages were placed on a 96-well membrane (5.7-mm diameter, 5-mm pore size; ChemoTX from Neuro Probe, Gaithersburg, MD) in RPMI-1640 containing 0.1% BSA (Sigma-Aldrich). The cells were allowed to migrate toward the chemotactic factors at the indicated concentrations for 60 min. Migrated cells were fixed (2% paraformaldehyde, VWR International AG, Dietikon, Switzerland) and stained with DAPI (Sigma-Aldrich), and migration was quantified as the total pixel count of DAPI-stained nuclei under the fluorescence microscope (one photo per membrane and two replicate wells per experiment). Migration indices were calculated over control values. The Formyl-Methionyl-Leucyl-Phenylalanine peptide (fLMF) (Sigma Aldrich) was used as a positive control.

### Characterization of Macrophages

To confirm the polarization of the resting macrophages into the M1 (INFγ, LPS) and M2 (IL-4, IL-13) subtypes following stimulation, the expression of different markers characteristic for the two subpopulations were measured (see Figure S1 and [14]). INFγ and LPS stimulation increased the mRNA expression of the pro-inflammatory cytokines TNFα and/or IL-1β and the cell surface expression of CD80. IL-4 and IL-13 stimulation increased the mRNA expression of the anti-inflammatory cytokine IL-10, reduced the mRNA of the pro-inflammatory IL-1β and TNFα, and increased the surface marker CD206.

### Statistical Analysis

Statistical analysis was performed with Microsoft Office Excel 2003 (Microsoft Corporation, Redmond, CA, USA). The level of FPR1 mRNA, protein and chemotaxis were compared using a two-sided t-test and a two-sided *p* value of <0.05 was considered significant.

**Figure 3:**
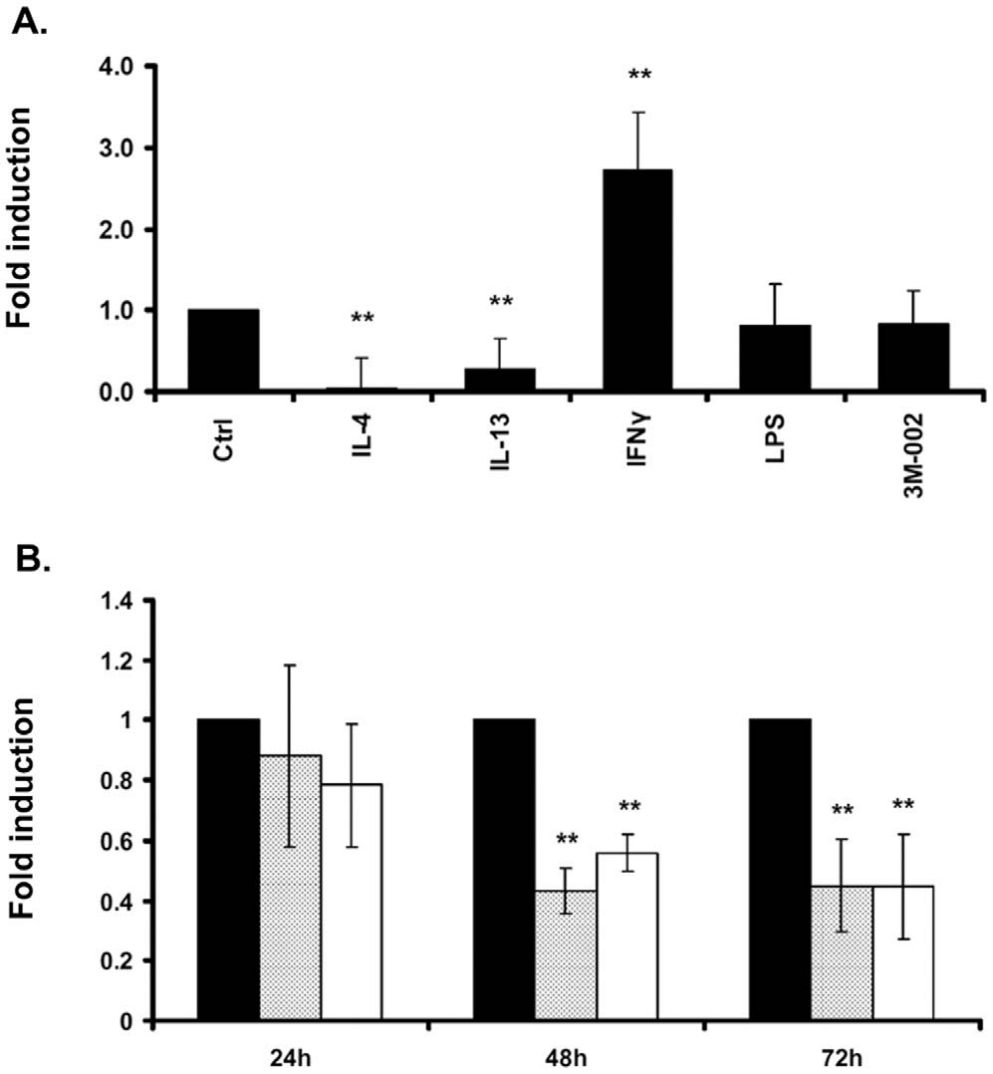
Regulation of FPR1 cell surface expression in human macrophages. FACS analysis was performed to investigate cell surface expression of FPR1 upon treatment with the indicated stimuli. A) FPR1 protein expression after stimulation of human macrophages for 48 hours with stimuli which were shown to regulate FPR1 mRNA expression (IL-4 (10 ng/ml), IL-13 (10 ng/ml), INFγ (50 ng/ml), LPS (100 ng/ml), 3M-002 (3 µM)). B) Time-course experiment of FPR1 protein expression in controls macrophages (black), or macrophages stimulated with IL-4 (10 ng/ml) (dotted) and IL-13 (10 ng/ml) (white) for 24, 48 and 72 hours. Values are presented relative to unstimulated macrophages. Bars display the mean and the standard deviation (±SD) of three independent experiments. *p<0.05, **p<0.01.

**Figure 4:**
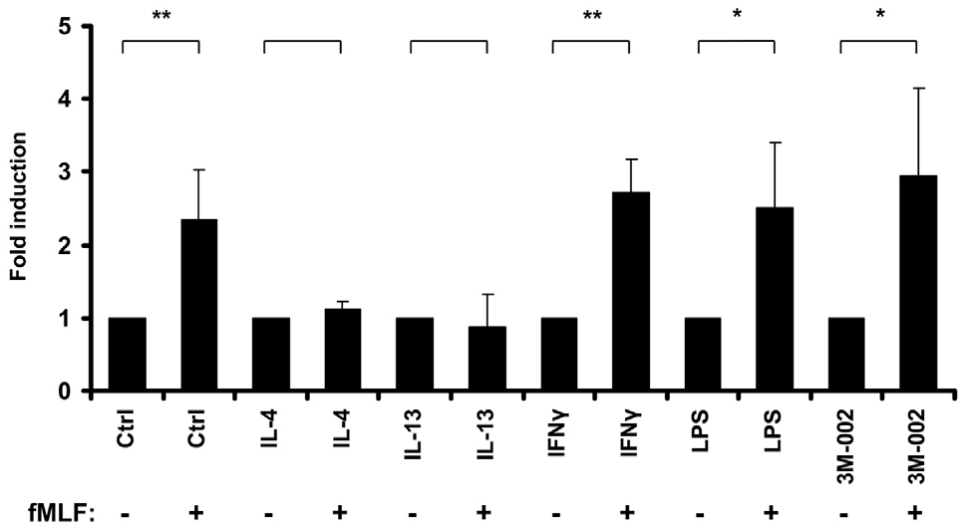
fLMF chemotaxis assays with human macrophages. Control (Ctrl) and IL-4 (10 ng/ml), IL-13 (10 ng/ml), IFNγ (50 ng/ml), LPS (100 ng/ml), and 3M-002 (3 µM) stimulated macrophages were allowed to migrate towards the chemotactic factor fLMF (+). Medium without chemotactic factor (−) was used as control for basal migration in each experiment. Graphs show the mean migration index compared to each individual control (n = 3) and error bars display the standard deviation (±SD). *p<0.05; **p<0.01.

## Results

Primary neutrophils, monocytes and resting macrophages express high levels of FPR1 on the cell surface (Figure 1). To investigate the regulation of the *FPR1* gene in leukocytes, primary human macrophages were stimulated with different pro- and anti-inflammatory cytokines or TLR agonists for 24 hours and the relative mRNA expression was measured by qPCR. Compared to the mRNA expression of resting macrophages, stimulation with IL-4 and IL-13 reduced FPR1 mRNA expression, while stimulation with IFNγ, LPS and with the TLR8 ligand 3M-002 further increased the FPR1 mRNA in macrophages (Figure 2A). No regulation of FPR1 mRNA levels was seen with IL-3, IL-1β, IL-6, TNFα, the TLR9 ligand CpG, the TLR3 ligand Poly I:C, and the TLR7 ligand 3M-001.

To corroborate the downregulation of the FPR1 mRNA following IL-4 and IL-13 stimulation, we measured the mRNA levels in a time course experiment for 72 hours (Figure 2B). Both cytokines reduced the FPR1 mRNA already after stimulation for 24 hours to less than 50% and after 48 hours to less than 20% of the level of resting macrophages (Figure 2B).

To investigate whether the regulation on the mRNA level translated into differential cell surface expression of FPR1, we analyzed the individually stimulated human macrophages for FPR1 cell surface expression by FACS analysis (Figure 3A). Similar to the results obtained on the mRNA level, we observed a decrease in FPR1 cell surface expression following stimulation of macrophages with IL-4 and IL-13 and an increase following IFNγ stimulation. However, no increase in FPR1 cell surface expression was observed following LPS and TLR8 (3M-002) stimulation, although both of these stimulations led to increased FPR1 mRNA levels. In addition we observed no increase in intracellular FPR1 staining following stimulation of macrophages with these stimuli (data not shown). In contrast, in a time-course experiment, IL-4 and IL-13 both led to sustained reduction in cell surface expression of FPR1 for up to 72 hours following stimulation (Figure 3B).

To show that the reduced cell surface expression of FPR1 in IL-4 and IL-13 stimulated human macrophages has functional relevance, we investigated chemotaxis of macrophages towards the FPR1 ligand fLMF (Figure 4). As expected from the cell surface expression of FPR1, resting macrophages migrated towards the chemotactic stimulus. In contrast, macrophages stimulated by IL-4 and IL-13, which have reduced mRNA and protein expression of FPR1, showed no chemotaxis towards fLMF. This indicates that IL-4 and IL-13 stimulated M2 macrophages will not respond to FPR1 ligands unlike resting macrophages or pro-inflammatory M1 macrophages stimulated with IFNγ or LPS.

## Discussion

We studied the regulation of FPR1 receptor expression in primary human macrophages and observed that neutrophils, monocytes and resting macrophages express the receptor on their cell surface. Polarization of macrophages towards a pro-inflammatory M1 macrophage further increased FPR1 mRNA levels but did not consistently increase protein expression or chemotaxis towards the FPR1 ligand fMLF. In contrast, polarization of primary human macrophages with IL-4 and IL-13 leading to the alternatively activated M2 macrophages, reduces FPR1 cell surface expression and abolishes chemotaxis towards fMLF. These results show that M2 macrophages will not react to triggering of FPR1, limiting the role for FPR1 to chemotaxis and superoxide production in resting and pro-inflammatory M1 macrophages.

Stimulation of FPR1 by TLR4 agonists has previously been observed in mouse macrophages and the mechanism involved in this increase has been investigated [15], [16]. These data demonstrated that FPR1 mRNA levels are dramatically elevated in both macrophages and neutrophils following exposure to LPS and that this resulted both from elevated transcription and from stabilization of the FPR1 mRNA [16]. In this line, higher binding of the fLMF peptide has been observed in leukocytes from patients suffering from Crohn’s disease or emphysema indicating that FPR1 expression on leukocytes may serve as a marker for systemic inflammation [17], [18]. Our data in human primary macrophages corroborate these findings and extend the number of stimuli leading to FPR1 upregulation by additionally identifying IFNγ and triggering of TLR8 as effective stimuli. IFNγ has been shown to induce effects resulting in heightened immune surveillance and immune system function during infection and to promote M1 macrophage polarization and activation [19]. In contrast, TLR8 signalling is thought to be rather specific for triggering immune responses via recognition of viral RNA in infected cells. TLR8 is one of pattern recognition receptors which is triggered by viral single stranded RNA and which was shown to stimulate macrophages to secrete IFNα and pro-inflammatory, as well as regulatory cytokines [20]. Since both of these stimuli increase FPR1 expression it is tempting to speculate that FPR1 may have a role in host responses to viral infections in addition to its proposed role in fighting bacterial infection. In this line the GP-41 envelope protein of the human immuno-deficiency virus (HIV) [21] and a secreted peptide from Herpes simplex virus-2 (HSV) [22] were shown to specifically activate the FPR1 receptor, and hence to modulate innate immunity during viral infection. However, whether FPR1 has a role in immune surveillance or whether HIV-1 and HSV-2 have evolved to desensitize the effector functions of FPR1, is not known [23].

While the pro-inflammatory stimulation of macrophages raises or at least conserves FPR1 cell surface expression, stimulation of macrophages with IL-4 and IL-13 abolishes FPR1 receptor expression and functionality in polarized M2 macrophages. Similar results were obtained on the mRNA level when mouse peritoneal macrophages were stimulated with IL-4 or IL-13 [24]. In these mouse macrophages, IL-4 and IL-13 suppressed the expression of FPR1 mRNA by a Stat6 dependent mechanism, which eliminated primary transcripts prior to maturation and depended on the constitutive instability of pre-existing FPR1 mRNA [24]. Our results expand these findings showing that also cell surface expression of the receptor and chemotaxis towards fMLF are abolished in M2 macrophages. Such a downregulation by the Th(2) cytokines is in line with the presumed role of FPR1 as a pro-inflammatory receptor supporting chemotaxis and superoxide production in leukocytes. While microbial peptides act as chemoattractants to recruit neutrophils to the site of challenge, where they phagocytose invading microorganisms to protect the host, the acute inflammation has a programmed fate to resolve inflammation upon clearance of the microorganisms to protect the host against unnecessary tissue damage [25]. Resolution of inflammation is part of this program to promote the homeostatic restoration of normal tissue structure and function, and IL-4 is a prototypic mediator of such activity which is known to promote the development of a Th(2) response [26], [27]. Downregulation of FPR1 in M2 macrophages indicates that FPR1 may not be necessary for the non-phlogistic alternative macrophage, clearing the inflamed tissue from apoptotic cells and microorganisms [28], [29].

In summary, we show that primary human neutrophils, monocytes and most polarized macrophages do express FPR1 on their cell surface, while IL-4 and IL-13 polarized M2 macrophages do not express functional FPR1 on their cell surface. These data argue for a role of FPR1 in resting and classically activated macrophages to support chemotaxis and superoxide production, while non-phlogistic alternative macrophages cannot use it.

## Supporting Information

Figure S1 **Measurement of the different M1 markers after stimulation of human macrophages with IFNγ (50 ng/ml)) for 48 hrs (surface marker CD80) or 24 hrs (relative expression of IL-1β and TNFα).** A: relative mRNA expression of IL-1β and TNFα normalized for GAPDH as fold induction of non-stimulated cells. B: FACS analysis of CD80 expression on non-stimulated cells (left panel) and on stimulated cells (right panel). Autofluorescence of the cells is shown in light grey, the isotype control in grey and cells labeled with CD80 antibody in black. MFl: median fluorescence intensity.
(TIF)

Table S1 Oligonucleotides used for quantitative RT-PCR.
(DOC)
