# Supplementary material for: Regulation of the Formyl Peptide Receptor 1 (FPR1) Gene in Primary Human Macrophages
Source: PLoS One. 2012 Nov 21;7(11):e50195. doi: 10.1371/journal.pone.0050195 (PMC3503994; doi:10.1371/journal.pone.0050195)
Supplement: Table S1 — Oligonucleotides used for quantitative RT-PCR. (DOC) [file pone.0050195.s002.doc]

**Supplementary Table S1**

| **Gene** |  | **Sequence from 5’ to 3’** | **Product length in bp** |
| --- | --- | --- | --- |
| **FPR1**  NM_001193306.1 | Upper  Lower | CTGAGTCACTCTCCCCAGGA  CCAGGAAGAGATAGCCAGCA | 189 |
| **GAPDH**  NM_002046.3 | Upper  Lower | CCCATGTTCGTCATGGGTGT  TGGTCATGAGTCCTTCCACGATA | 145 |
